# Progress of Obstetrics and Gynæcology

**Published:** 1874-06

**Authors:** A. Reeves Jackson

**Affiliations:** Lecturer on Gynæcology, Rush Medical College, Chicago


ARTICLE II.—*Progress of Obstetrics and Gynæcology.* By A. REEVES JACKSON, M.D., Lecturer on Gynæcology, Rush Medical College, Chicago.

1. Is Ovulation the Sole Cause of Menstruation? By C. C. MATTESON, M.D. (*Amer. Supplement to Obstetrical Jour. of Great Britain and Ireland*, April, 1874.)

2. On the Necessity for Caution in the Employment of Intra-Uterine Stems. By Dr. EDIS. (*London Lancet*, April, 1874.)

3. On the Prevention of Uterine Inflammation. By Dr. EDWARD J. TILT. (*Half-Yearly Abstract of the Medical Sciences*, Jan. 1874. *British Med. Journal.*)

1. The question treated by Dr. Matteson involves the correctness of what is known as the ovulation theory of menstruation. Since the enunciation of this theory by Dr. John Power, in 1821, physiologists have, one by one, given it their adherence, and so generally is it now approved, that it has come to be regarded as heterodox to dispute its truth. Occasional attacks, it is true, have been made upon it, but they have thus far been unsuccessful.

Certainly it is not a hopeful task to attempt anything like a refutation of the correctness of the matured opinions of such men as Raciborski, Pouchet, Bischoff, Baer, Barry, Robert Lee, Tyler Smith, Chas. D. Meigs, Cazeaux and Leishman, all of whom are mentioned by Dr. Matteson as fully endorsing the ovular theory; and yet one author does make the attempt, not by elaborate argument, but simply by the presentation of certain stubborn facts.

One of the strong arguments made use of by the adherents of the ovulation theory is the asserted fact that the removal of both ovaries from a woman causes an invariable and immediate cessation of menstruation. The aim of Dr. Matteson is to show that menstruation has taken place in women who have undergone the operation alluded to, "and therefore that ovulation cannot be the *sole* cause of that phenomenon."

In order to sustain this position, he has industriously collected the notes of ten cases in which, after double ovariotomy, the patients had continued, with more or less regularity, to have a sanguineous discharge, attended by the ordinary symptoms of the menstrual flow, recurring in some instances for a few months, and in others for many years.

Dr. Matteson does not think that the influence of "habit," to which the discharge was attributed in the first reported cases of the kind mentioned, is rationally applicable to those in which it has continued to occur regularly through nine and ten years, however deserving of consideration it might be in those incomplete ones where the flux appeared but once or twice. He adds, "What then remains? Can we fail to admit, with our present knowledge, that menstruation does take place when the ovaries are wanting?"

2. The paper of Dr. Edis was read recently at a meeting of the Obstetrical Society of London. Its object was to forcibly impress the necessity of caution in the use of intra-uterine stems, on the part of those who regarded this mode of treatment as necessary. He detailed several cases illustrating the dangers which may arise from the use of the instrument. In order to lessen these dangers, he advised preparatory treatment and the supervision of patients wearing intra-uterine stems.

The paper is certainly a most timely one. When, a few years ago, the late Prof. Simpson introduced to the profession his so-called galvanic intra-uterine stem pessary, with all the enthusiasm that belonged to his nature, the instrument became at once largely employed both in Great Britain and on the Continent of Europe. But so indiscriminately were the cases selected—or rather, there was such an entire lack of selection—that many accidents, some of them of a fatal character, soon resulted from this mode of treatment. The consequence was that it was discarded as suddenly as it had come into use. Nevertheless, it had in so many cases been productive of benefit, that gynæcologists again began to regard it with favor, and with increased care in its employment, the instrument has come to be once more looked upon as a legitimate and useful therapeutic means. Indeed, there is evidently a rapidly growing tendency to resort to it for the relief of all cases of flexion productive of morbid symptoms, for dysmenorrhœa dependent upon this or other obstructive cause, and for cases of defective uterine development. However, the cases recorded by Dr. Edis and other observers, show that still greater care must be taken, or a valuable remedial agent may again fall into disrepute.

During the discussion which followed the reading of Dr. Edis' paper, Dr. Savage remarked on the capriciousness of uterine tolerance, and thought that we had yet to acquire the art of bringing the uterus to a condition which would insure immunity from danger when subjected to treatment.

For ourselves, we think that intra-uterine stems of any kind, whether of metal or of vulcanite, rigid or flexible, should never be used: 1. During the continuance of active congestion or inflammation of the womb or its mucous lining; 2. If there be perimetric inflammation or its results, as adhesion or deposits; 3. No force whatever should be used in their introduction; 4. The patient should be seen daily for the first week, and subsequently at intervals of three or four days. If these precautions be duly observed, this mode of treatment, which in our opinion is the most scientific, most useful, and, all things considered, the *best* for certain abnormal conditions of the uterus, may be used with at least as much safety as attends the use of most surgical means.

3. In this paper the author calls attention to the fact that the most frequent causes of uterine inflammation are to be found in the conditions attending parturition and abortion; and his own experience leads him to believe that metritis, in some form, is an almost inevitable sequel of a tedious labor or a bad miscarriage, although in some instances years may elapse before the disease is recognized. It is well known to obstetricians that most cases of tedious and instrumental labor are attended by contusion and laceration of the cervix. These lacerations, if not very extensive, ordinarily heal readily provided the woman be healthy; but if the injuries be extensive, or the woman be in feeble health, they may give rise to very intractable forms of uterine inflammation.

A still more frequent cause of inflammation and other diseased conditions of the womb, is the defective involution of the organ. Both physician and patient regard parturition as a natural function. This is of course correct, but an exaggerated belief in its consequent safety has too frequently the effect of leading them to neglect the timely use of means for ascertaining accurately the existence and extent of organic lesions that retard recovery after labor or abortion. One of the results of this erroneous practice is the non-recognition of subinvolution until it has become chronic and more difficult to cure. If the damage done to the womb by parturition were rightly appreciated, measures calculated to prevent injurious results following the process would be more frequently employed.

Whenever, after four or five weeks of careful nursing and proper hygienic surroundings, a woman still continues weak, with persistent pains in the back, and red or muco-purulent vaginal discharge, a careful examination should be made, and nature should no longer be blindly trusted. Such examination will frequently reveal the existence of some of those structural lesions which cannot be cured without the aid of surgery.

Abortions are even more likely to be followed by inflammatory disorders than labor at full term. This results from the fact that women usually regard such accidents as of trivial importance, and take little subsequent care of themselves, whereas, in the opinion of the author, a month of convalescence is not too much to exact after a moderately bad miscarriage.

